# Pathobiome driven gut inflammation in Pakistani children with Environmental Enteric Dysfunction

**DOI:** 10.1371/journal.pone.0221095

**Published:** 2019-08-23

**Authors:** Najeeha T. Iqbal, Sana Syed, Furqan Kabir, Zehra Jamil, Tauseef Akhund, Shahida Qureshi, Jie Liu, Jennie Z. Ma, Shan Guleria, Andrew Gewirtz, Christopher P. Duggan, Molly A. Hughes, Kamran Sadiq, Asad Ali

**Affiliations:** 1 Department of Pediatrics and Child Health, Aga Khan University, Karachi, Pakistan; 2 Department of Biological & Biomedical Sciences, Aga Khan University, Karachi, Pakistan; 3 Department of Pediatrics, University of Virginia, Charlottesville, VA, United States of America; 4 Department of Medicine, University of Virginia, Charlottesville, VA, United States of America; 5 Center for Inflammation Immunity & Infection, Georgia State University, Atlanta, Georgia, United States of America; 6 Division of Gastroenterology, Hepatology and Nutrition, Boston Children’s Hospital, Boston, Massachusetts, United States of America; 7 Departments of Global Health and Population, and Nutrition, Harvard T.H. Chan School of Public Health, Boston, Massachusetts, United States of America; New York State Department of Health, UNITED STATES

## Abstract

Environmental Enteric Dysfunction (EED) is an acquired small intestinal inflammatory condition underlying high rates of stunting in children <5 years of age in low- and middle-income countries. Children with EED are known to have repeated exposures to enteropathogens and environmental toxins that leads to malabsorptive syndrome. We aimed to characterize association of linear growth faltering with enteropathogen burden and subsequent changes in EED biomarkers. In a longitudinal birth cohort (n = 272), monthly anthropometric measurements (Length for Age Z score- LAZ) of asymptomatic children were obtained up to 18 months. Biological samples were collected at 6 and 9 months for the assessment of biomarkers. A customized TaqMan array card was used to target 40 enteropathogens in fecal samples. Linear regression was applied to study the effect of specific enteropathogen infection on change in linear growth (ΔLAZ). Presence of any pathogen in fecal sample correlated with serum flagellin IgA (6 mo, r = 0.19, p = 0.002), fecal Reg 1b (6 mo, r = 0.16, p = 0.01; 9mo, r = 0.16, p = 0.008) and serum Reg 1b (6 mo, r = 0.26, p<0.0001; 9 mo, r = 0.15, p = 0.008). At 6 months, presence of Campylobacter [β (SE) 7751.2 (2608.5), p = 0.003] and ETEC LT [β (SE) 7089.2 (3015.04), p = 0.019] was associated with increase in MPO. Giardia was associated with increase in Reg1b [β (SE) 72.189 (26.394), p = 0.006] and anti-flic IgA[β (SE) 0.054 (0.021), p = 0.0091]. Multiple enteropathogen infections in early life negatively correlated with ΔLAZ, and simultaneous changes in gut inflammatory and permeability markers. A combination vaccine targeting enteropathogens in early life could help in the prevention of future stunting.

## Introduction

Environmental Enteric dysfunction (EED) is a subclinical inflammatory disease of the small intestine characterized histologically by blunted villi, elongated crypts and increased lymphocytic infiltration of the lamina propria [1, 2]. These histological changes are associated with subsequent malabsorption, impaired cognitive development [3, 4], reduced responsiveness to nutritional intervention [5], and reduced immunogenicity of oral vaccine [6, 7] in apparently healthy children [8, 9]. The main impediment in diagnosis of EED is examination of upper bowel mucosa, which is impractical for a high proportion of children failing nutritional intervention [10]. The role of enteropathogens are well documented in studies such as Etiology, Risk Factors and Interactions of Enteric Infection and Malnutrition and the Consequences for Child Health and Development (MAL-ED) and Global Enteric Multicenter Study (GEMS) [11, 12]. However, asymptomatic enteropathogen carriage in children with EED has not been studied in detail with growth faltering and change in EED biomarkers. The knowledge of gut pathobiome in apparently healthy children may be associated with the causal pathway of EED, which is a multifactorial disease process accompanied by repeated exposure to pathogens and possible dysbiosis of microbiome during infancy [13]. In developing countries, it is known that in the absence of diarrhea, 15% of children under five years are infected with ETEC as asymptomatic carriers [14].

In the current EED cohort, we first examined the relationship of bacterial translocation marker along with gut and systemic inflammatory biomarkers in children with growth faltering [15]. The presence of IgA against bacterial flagellin and LPS was found to be associated with enteric inflammation and with subsequent decline in linear growth. In order to explore the contribution of enteropathogens in EED, we further characterized the association of enteropathogen burden with putative biomarkers and subsequent growth faltering at 18 months of age. We hypothesized that increased enteropathogen burden in the early period of life is associated with future decline in LAZ scores and corresponding changes in EED biomarker profiles. Identification of any single or a group of enteropathogens could provide early screening of children who are at risk of developing EED.

## Material and methods

### Ethics statement

Institutional approval was granted by the Aga Khan University Ethical Review Committee (ERC# 2446 Ped ERC 13) and the University of Virginia Institutional Review Board. All parents provided written informed consent for participation of their children in the study.

### Study design and participants

Subjects included in this analysis were part of a prospective community-based active surveillance birth cohort and were followed longitudinally for anthropometrics and biomarker measurements, response to Ready-to-Use-Therapeutic-Food (RUTF) and subsequent endoscopic/ histopathological examination in cases of inadequate growth response to RUTF [16, 17]. Additional descriptive characteristics of the study participants are shown in S1 Table.

Newborns (n = 272) were enrolled and assessed during routine surveillance of pregnant women of reproductive age (13–49 years) by community health workers (CHWs) [18]. Study inclusion criteria were: 1) newborns aged up to 14 days; 2) absence of any major congenital abnormalities and; 3) ability to obtain informed consent from parents or guardians. Infants of families planning to move out of the study area within 6 months of birth were excluded from the trial. Enrolled children were followed from birth (0 to 14 days) until 18 months of age with weekly home visits during the study period from October 2012 to November 2015 for diarrheal and acute respiratory infection episodes (S1 Fig). All families enrolled in the study were provided with cell phone contact information of key study physicians to enable direct and immediate contact in the case of any urgent medical need. Monthly measurements were recorded by trained Community Health Workers using standard techniques: child’s weight using a digital infant balance with 20-g precision (TANITA 1584) and child’s length using a rigid length board with a movable foot piece with 1 mm precision. Standardization of measurements was ensured through regular staff training and cross checks.

### Biological specimen collection

Blood was obtained from enrolled children at 6 and 9 months of age. Samples were centrifuged in the field site research laboratory and plasma was removed within 2 h of blood collection. Samples were transported at 4°C from the field site at Matiari, Pakistan to the Aga Khan University Infectious Disease Research Lab (IDRL) under cold chain maintenance. Aliquots were stored at -80°C.

For collection of fecal samples, the mother/care-taker was instructed to use a diaper provided by the research staff that was lined with a thin plastic sheet to prevent absorption. Defecated samples were collected from the participant’s homes. Using a clean spatula, the fecal samples were transferred to a clean container. The fecal samples were transported from the child’s home to a peripheral laboratory in a 15 liter Coleman cooler with cold chain maintenance at 4°C. The samples were further aliquoted into small vials and were stored in the central laboratory IDRL at - 80ºC.

#### Measurement of biomarkers

Peripheral blood samples were tested for flagellin and LPS-specific IgA and IgG concentrations measured by ELISA as previously reported [19]. Microtiter plates were coated with purified *E*. *coli* flagellin (100ng/well) or purified *E*. *coli* LPS (2 μg/well). Serum samples were diluted 1:200 and applied to the coated wells. After incubation and washing, the wells were incubated with anti-human IgA (KPL) or IgG (GE Healthcare) coupled to horseradish peroxidase. Quantification of total immunoglobulin was performed using the colorimetric peroxidase substrate tetramethylbenzidine (TMB) and read at 450 nm optical density (OD) on an ELISA plate reader. Data was reported as OD corrected by subtracting background levels, which were determined by reading in samples lacking serum. Commercial ELISA kits were used for the estimation of regenerating gene 1β (Reg 1b) (TechLab, Blacksburg, Virginia) in feces and serum. For intestinal inflammation, Myeloperoxidase (MPO) kit (Immunodiagnostic AG, Stubenwald-Allee, Bensheim) and Neopterin (NEO) (GenWay Biotech, San Diego, CA) were used for fecal samples as reported previously [20]. Biomarkers of systemic inflammation (C-reactive protein [CRP], alpha- 1-acid glycoprotein [AGP], and ferritin) were analyzed on the Hitachi 902 analyzer (Roche Diagnostics, Holliston, MA). All protocols were followed as per manufacturers’ instruction. The final dilution for serum and fecal biomarkers was determined by selecting the most appropriate concentration of a biomarker falling in the linear range of standard curve. Reg 1b was performed in two dilutions of 1:40,000 and 1:100,000, NEO at the dilution of 1:250 and MPO at 1:500. All plates were read on the Biorad iMark (Hercules, CA) plate reader.

#### TAC analysis of fecal samples

TaqMan Array card (TAC): The TaqMan low density array card or TAC allows molecular detection of multiple enteric pathogens using a customized detection platform based on a real time PCR detection system [21]. The enteric pathogen panel included viruses, bacteria and helminths. The TAC card was customized to detect microbial pathogens in the fecal samples collected at 6 months (n = 272) and 9 months (n = 271). This platform has been successfully used in our lab for multicenter studies such as GEMS and MAL-ED [22, 23]. Briefly, total nucleic acid (TNA) was extracted from approximately 180-220g of fecal samples using the bead beating method by adding 370mg of glass beads (Sigma, Aldrich, UK) followed by TNA extraction using QIAmp DNA Stool MiniKit (Qiagen,Germantown, MD). As per protocol, all samples were spiked with internal controls of PhHV (Phocine Herpes Virus) and MS2 (MS2 bacteriophage) as DNA and RNA targets respectively for validation of samples and to check the efficiency of extraction, reverse transcription and amplification steps. The TAC protocol was modified from the Next Generation Molecular Diagnostic project (Houpt Lab, University of Virginia, Charlottesville, USA). 100μl of TNA was eluted from the DNA extraction kit. Of this, 40 μl was mixed with 60μl of AgPath one step RT-PCR kit (Ambion, Applied Biosystem) and 100μl was loaded on a TAC card through microfluidic ports. The card was sealed and processed on ViiA7 (Applied Bio systems, Thermofisher, USA). A total of 8 samples were run in a single card along with extraction blank (consisting of nuclease free water with PhHV & MS2) and PCR blank (consisting of nuclease free water only). The samples were considered valid positive if target Ct value was less than 32, reference extraction blank was negative for target and internal control, and MS2 had Ct value less than 38.

These TAC cards were customized to detect 40 common enteropathogens including the following: Adenovirus, Aeromonas, *Ancylostoma*, *Ascaris*, Astrovirus, *Bacteroides fragilis*, *Campylobacter jejuni* and *Campylobacter coli*, *Clostridium difficile*, Cryptosporidium hominis and Cryptosporidium parvum, *Cyclospora*, *Encephalitozoon intestinalis*, *Entamoeba histolytica*, *Enterocytozoon bieneusi*, enteroaggregative *Escherichia coli* (EAEC), enteroinvasive *E*. *coli* (EIEC), enteropathogenic *E*. *coli* (EPEC), enterotoxigenic *E*. *coli* (ETEC), shigatoxigenic serotypes of *E*. *coli* (STEC), enterovirus, *Giardia*, *Helicobacter pylori*, *Isospora*, *Mycobacterium tuberculosis*, *Necator*, norovirus, rotavirus, *Salmonella*, sapovirus, *Shigella*, *Strongyloides*, *Trichuris*, and *Vibrio cholerae*.

### Statistical analysis

WHO Child Growth Standards (WHO Anthro, Geneva, Switzerland) [24] was used to calculate z-scores and assess growth both as continuous measures [length-for-age z-score (LAZ), weight-for-age z-score (WAZ) and weight-for-height z-score (WHZ)] and as categorized variables of stunting [LAZ < −2 SD (standard deviation), underweight as WAZ < −2 SD and wasting as WHZ < −2 SD]. In accordance with WHO recommendations, we excluded outliers of LAZ (<-6 or >6), WHZ (<-5 or >5), and WAZ (<-6 or >5) [25].

Effect of specific enteropathogen infection on children’s length over time was measured by using delta LAZ, a change in length-for-age (ΔLAZ = 18months-birth). The relationship between the change in z-scores (LAZ/WAZ/WHZ) and infection with specific pathogens at 6 and 9 months was modeled via simple linear regression to examine the unadjusted association of enteric infection (categorical variable, either yes or no for each pathogen) with the continuous outcome of change in LAZ score from birth to 18 months. Linear regression was used to further evaluate the association between ΔLAZ (dependent variable) with specific pathogens infection (independent variables), adjusting for antibiotics use:

$$Model\;1:\;\Delta LAZ=\beta_{0}+\beta_{1}\;pathogen+\beta_{2}antibiotics+\varepsilon$$

**Fig 1:**
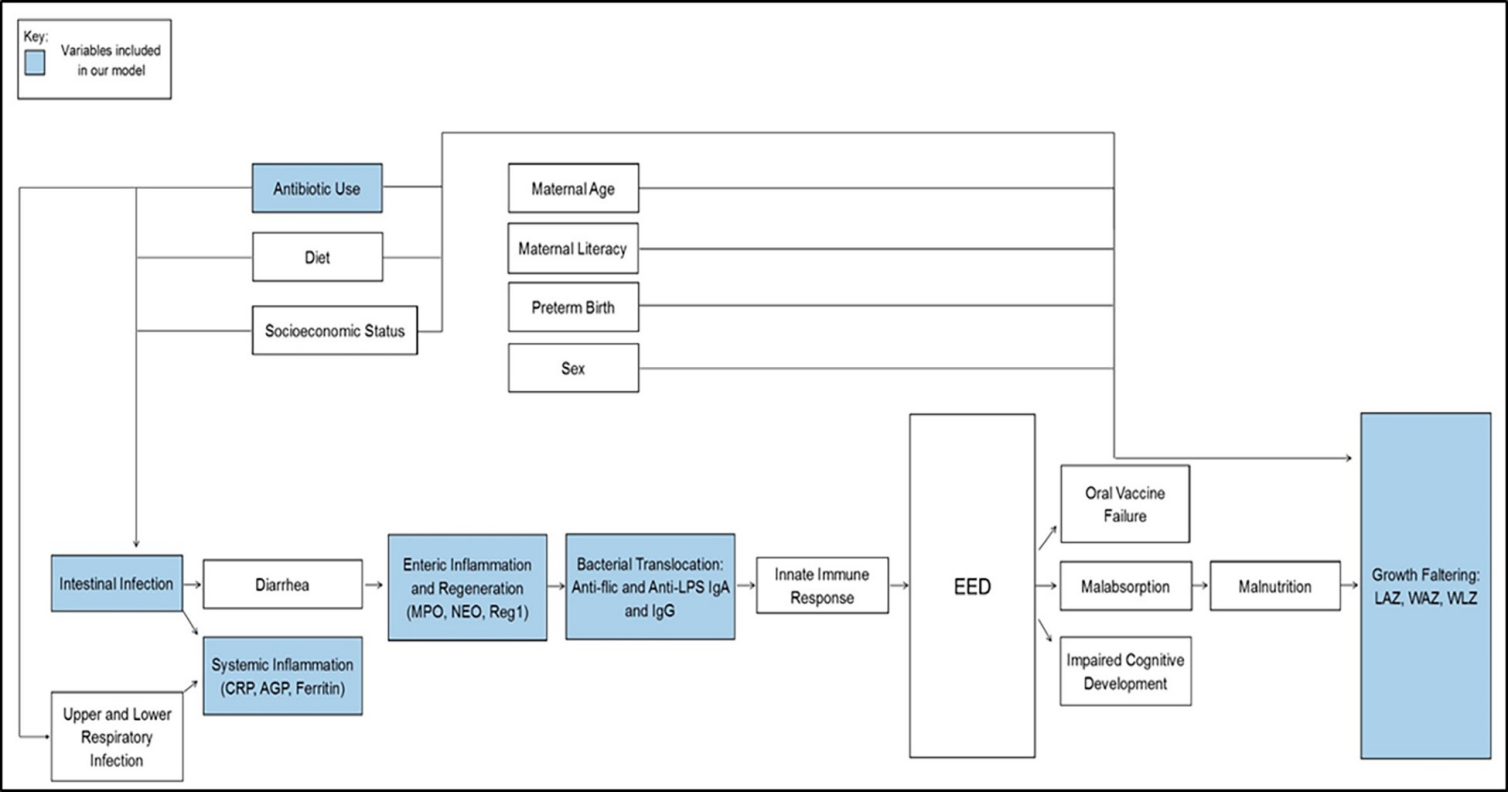
Hypothesized causal pathways for environmental enteric dysfunction (EED). Analyses included factors highlighted in blue in order to model the relationship between fecal enteropathogens and markers of bacterial translocation, inflammatory biomarkers with growth faltering in association with EED.

Where β is the estimated regression coefficient, ε is the error term, “pathogen” (categorical variable) refers to whether or not each specific pathogen has infected a given child (a different term for each pathogen in the model), and “antibiotics” (categorical variable) refers to the use of antibiotic at least once during the period of 6 or 9 months as “Yes”. Our approach was informed by a hypothesized conceptual causal diagram (Fig 1) for EED determinants. Correlations were calculated using Pearson correlation coefficient with associated 95% confidence between positive counts of pathogens (continuous variable) and ΔLAZ. Similar correlations were calculated between positive counts of pathogens (continuous variable) and our biomarkers of systemic inflammation (CRP, AGP, ferritin), enteric inflammation and intestinal regeneration (MPO, NEO, Reg 1b feces and serum), and bacterial translocation (anti-flic and anti-LPS IgA and IgG).

Biomarkers found to be significantly correlated with increasing pathogen counts were taken as part of a sub-analysis in which we used linear regression models at 6 months (anti-flic IgA, MPO, Reg 1b feces and serum) and 9 months (CRP, AGP, Reg 1b feces and serum) with each biomarker as the dependent variable and infection with specific pathogens as independent variables (categorical variable) with antibiotic use included as a covariate:

$$Model\;2:\;Biomarker=\beta_{0}+\beta_{1}\;pathogen_{1}+\beta_{2}pathogen_{2}+\ldots +\beta_{k}pathogen_{k}+\beta_{k+1}antibiotics+\varepsilon$$

Where β is the estimated regression coefficient, ε is the error term, “pathogen_k_” (categorical variable) refers to whether or not each specific pathogen has infected a given child (a different term for each pathogen in the model), and “antibiotics” (categorical variable) refers to the use of antibiotics at 6 and 9 months. This multivariable analysis was performed for each biomarker. All analyses were performed using SAS version 9.4 (SAS Institute, Cary, NC, USA).

## Results

### Impact of enteropathogens burden on LAZ

Table 1 shows the proportions of children infected with different pathogen subtypes and their respective mean LAZ scores. In each category of enteropathogen that included bacteria, viruses and protozoa, no difference was found in the mean LAZ score in children with or without particular infection. This trend was similar at both 6 and 9 months.

**Table 1:** Characteristics of the Pakistani cohort at 6 months (N = 272) and 9 months (N = 271) with regards to pathogen infection and length-for-age Z (LAZ) scores.

	Enteropathogens at 6 months									Enteropathogens at 9 months								
	Bacteria			Protozoa			Viruses			Bacteria			Protozoa			Viruses		
	Yes	No	p^¥^	Yes	No	p^¥^	Yes	No	p^¥^	Yes	No	p^¥^	Yes	No	p^¥^	Yes	No	p^¥^
N (%)	247(90.1)	27(9.9)	0.93	137(50.0)	137 (50.0)	0.70	249 (92.9)	25 (7.1)	0.099	256(93.8)	17(6.2)	0.098	194(71.1)	79(28.9)	0.42	253 (92.7)	20(7.3)	0.35
LAZ Mean (SD)	-2.73 (1.08)	-2.70(1.67)		-2.75(1.15)	-2.70 (1.14)		-2.69 (1.16)	-3.09 (0.90)		-2.76(1.15)	-2.28(0.97)		-2.77(1.18)	-2.64(1.05)		-2.75(1.16)	-2.50(0.87)	

Notes: “Yes” = infection with 1 or more pathogens of the specified category. “No” = no infections of the specified category. LAZ18 = length-for-age Z score at the 18th months of life. None of the differences in LAZ score between children infected or not infected with pathogens of a given category were significant. p^¥^ (p-value) refers to the difference between mean LAZ scores at 18 months of age in the groups positive and negative for enteropathogens at 6 or 9 months.

### Association of enteropathogen counts with ΔLAZ score and inflammatory biomarkers

Overall, the frequency of bacterial infection was found to be similar in the samples collected at 6 and 9 months with the exception of Campylobacter and Cryptosporidium. Among viral and protozoal pathogens, Adenovirus, Sapovirus, Giardia and Cryptosporidium frequencies were higher at 9 months compared to 6 months (S2 Fig)

Table 2 shows correlation coefficients between growth and enteropathogen burden. There was a significant negative correlation between enteropathogen counts of all subtypes at 6 months and ΔLAZ (18 months-birth). This effect was no longer significant at 9 months. We further analyzed positive counts of enteropathogen with putative EED biomarkers. Presence of enteropathogen (all positive for bacteria, virus, protozoa) correlated with serum flagellin (flic) IgA, fecal Reg 1b and serum Reg 1b. Infection with bacteria correlated with MPO, protozoa with CRP, AGP and Ferritin, while viral infection with CRP.

**Table 2:** Correlation coefficient matrix of biomarkers^¥^ and the change in Z scores for length over the first 18 months of life with the number of enteropathogens infecting children at 6 and 9 months.

		Enteropathogens at 6 months				Enteropathogens at 9 months			
		Counts of All Positive Pathogens	Counts of Positive Bacteria	Counts of Positive Protozoa	Counts of Positive Viruses	Counts of All Positive Pathogens	Counts of Positive Bacteria	Counts of Positive Protozoa	Counts of Positive Viruses
**Growth**	ΔLAZ	-0.21*	-0.12	-0.15*	-0.15*	-0.062	-0.017	-0.036	-0.075
**Bacterial Translocation**	Anti-LPS IgA, OD	0.098	0.04	0.077	0.091	-0.0078	-0.046	-0.013	0.067
	Anti-LPS IgG, OD	0.036	0.09	-0.049	-0.0095	-0.030	0.011	-0.041	-0.043
	Anti-flic IgA, OD	0.19*	-0.0017	0.22**	0.23**	0.0026	-0.055	0.013	0.078
	Anti-flic IgG, OD	0.039	0.051	0.012	-0.0053	-0.052	-0.056	-0.048	0.019
**Systemic Inflammation**	CRP, mg/L	0.085	0.11	0.0072	0.011	0.066	0.075	0.16*	-0.13*
	AGP, mg/dL	0.018	0.040	0.016	-0.039	0.088	0.029	0.18*	-0.030
	Ferritin, ng/mL	-0.093	-0.11	0.069	-0.10	0.061	-0.036	0.19*	0.0066
**Enteric inflammation & regeneration**	MPO, ng/mL	0.083	0.15*	0.024	-0.076	0.045	0.020	0.042	0.028
	NEO, nmol/L	0.024	-0.015	0.013	0.067	-0.075	-0.057	-0.099	0.018
	Reg 1b Serum, ng/mL	0.26***	0.28***	0.12*	0.035	0.15*	0.16*	0.028	0.055
	Reg 1b fecal, μg/g	0.16*	0.16*	0.10	0.015	0.16*	0.11	0.10	0.082

The following were studied as continuous variables: Counts of all positive pathogens, counts of positive bacteria, counts of positive protozoa, counts of positive viruses and all biomarker concentrations.

Note: Values are Pearson correlation coefficients.

***p-value<0.0001

**p-value<0.001

*p-value<0.05.

Abbreviations: Flagellin = flic; Immunoglobulin = Ig; Lipopolysaccharide = LPS; alpha glycoprotein = AGP; C-reactive protein = CRP; Myeloperoxidase = MPO; Neopterin = NEO; regenerating gene 1 beta = Reg 1b, change in length-for-age Z scores over the first 18 months of life = ΔLAZ. ¥Biomarkers measured were indicative of systemic inflammation, bacterial translocation, enteric inflammation, or intestinal regeneration (left column indicates which biomarkers fall into each category).

### Infection with enteropathogens is associated with change in LAZ score

Table 3 shows the association of ΔLAZ^(18month-birth)^ with specific enteropathogen at 6 and 9 months of age. We found a nominally significant decline in LAZ with the positivity of Astrovirus, *Campylobacter*, Cryptosporidium and *Giardia* at 6 months (nominal p-value < 0.05). The effects of these enteropathogens on ΔLAZ became insignificant at threshold of 0.05 after the adjustment for false discovery rate (FDR). Nevertheless, *Campylobacter*, Cryptosporidium and *Giardia* were marginally associated with ΔLAZ at FDR threshold of 0.1. There was minimal or no change in delta LAZ for the given pathogens at 9 months compared to those at 6 months after FDR adjustment, even if Norovirus at 9 months was significantly associated with ΔLAZ at nominal significance level.

**Table 3:** The association of selected enteropathogens [at 6 (n = 272) and 9 (n = 271) months] with changes in LAZ scores over the first 18 months of life.

	Enteropathogens at 6 months				Enteropathogens at 9 months			
			p-values				p-values	
	β for ΔLAZ	SE	nominal	FDR-adjusted	β for ΔLAZ	SE	nominal	FDR-adjusted
Adenovirus 40/41	-0.05	0.25	0.856	0.856	-0.11	0.19	0.599	0.982
Astrovirus	-0.38	0.19	**0.046**	0.116	0.07	0.21	0.725	0.982
*Bacteroides fragilis*	0.33	0.21	0.121	0.192	-0.08	0.18	0.681	0.982
*Campylobacter jejuni/coli*	-0.36	0.16	**0.025**	0.091	-0.15	0.15	0.294	0.874
*Campy_pan*	-0.36	0.15	**0.014**	0.090	-0.10	0.14	0.455	0.982
Cryptosporidium	-0.45	0.20	**0.028**	0.091	-0.004	0.15	0.982	0.982
*Cyclospora*	0.26	0.66	0.688	0.688	0.44	0.36	0.228	0.874
EAEC	-0.21	0.15	0.180	0.225	-0.14	0.17	0.420	0.982
EIEC *Shigella*	-0.41	0.31	0.191	0.225	-0.03	0.24	0.902	0.982
EPEC	-0.004	0.14	0.979	0.979	0.003	0.14	0.982	0.982
ETEC_LT	-0.03	0.16	0.842	0.842	0.23	0.16	0.146	0.788
ETEC _STh	-0.54	-1.6	0.107	0.192	0.06	0.27	0.820	0.982
ETEC_STp	-0.05	0.27	0.846	0.846	-0.37	0.27	0.158	0.788
*Enterocytozoon bieneusi*	0.65	0.33	0.054	0.116	-0.013	0.21	0.951	0.982
Enterovirus	-0.17	0.16	0.278	0.301	-0.11	0.16	0.517	0.982
*Giardia*	-0.57	0.23	0.013	0.090	-0.36	0.19	0.055	0.546
Norovirus GI&II	-0.22	0.15	0.133	0.192	-0.29	0.15	0.050	0.546
Rotavirus	0.21	0.26	0.426	0.426	-0.13	0.33	0.702	0.982
STEC Stx1 stx2	0.30	0.44	0.494	0.494	0.10	0.32	0.748	0.982
Sapovirus	-0.14	0.25	0.565	0.565	-0.16	0.15	0.306	0.874

Notes: This regression model has been described in the Methods section as Model 1: ΔLAZ (18mo-birth) = β_0_ + β_1_ pathogen + β_2_ antibiotics + ε. The β_1_ is the estimated effect of each pathogen obtained from a linear regression model using ΔLAZ score as the dependent variable (continuous) and the presence of each pathogen (categorical) as the independent variable, adjusting for the antibiotic use (categorical variable) at 6 or 9 months. ΔLAZ refers to the change in length-for-age Z scores over the first 18 months of life. Nominal p-values were directly estimated from the regression, while FDR-adjusted p-values were calculated using Proc MULTTEST in SAS to account for multiple comparisons.

### Infection with specific enteropathogens is associated with an increase in inflammatory biomarkers

In order to analyze the effect of enteropathogen infection on inflammatory biomarkers, we selected biomarkers based on their significant correlation with increasing enteropathogen count (Table 2). The significant associations at 6 months included anti-flic IgA with astrovirus infection, anti-flic IgA with STEC (stx1, stx2) infection, MPO with *Campylobacter* infection, serum Reg 1b with ETEC ST infection, fecal Reg1 β with *Giardia* (Table 4). At 9 months, only serum Reg 1b reported significant correlation with *Campylobacter* infection (S2 Table).

**Table 4:** The association of select enteropathogens at 6 months (n = 272) with levels of specific biomarkers¥ at 6 months.

Enteropathogens	Biomarkers at 6 months											
	Anti-flic IgA			MPO			Reg1b Serum			Reg1b fecal		
	β	SE	p-value	β	SE	p-value	β	SE	p-value	β	SE	p-value
*Aeromonas*	-0.037	0.083	0.6540	-3939.221	10181.900	0.6992	-42.499	68.685	0.5366	-25.243	107.376	0.8143
*Bacteroides fragilis*	0.046	0.032	0.1521	-1370.069	3815.262	0.7198	48.266	26.182	0.0664	-20.334	40.886	0.6194
*C*. *difficile*	0.162	0.095	0.0888	15637.007	11575.970	0.1779	39.208	78.085	0.6160	-138.291	122.126	0.2586
*Campylobacter*	-0.038	0.021	0.0750	7751.217	2608.554	0.0032*	5.972	17.717	0.7363	47.283	27.842	0.0907
EAEC	0.010	0.023	0.6525	3825.984	2765.798	0.1678	-1.241	18.999	0.9480	-32.145	29.305	0.2737
EIEC *Shigella*	0.000	0.048	0.9943	1174.943	5827.794	0.8404	66.294	39.374	0.0935	105.623	61.463	0.0869
EPEC	0.026	0.022	0.2287	-3996.395	2666.754	0.1352	51.733	18.301	0.0051*	7.466	28.322	0.7923
ETEC LT	0.061	0.025	0.0140	7089.216	3015.043	0.0195*	34.956	20.396	0.0878	30.101	31.852	0.3456
ETEC ST	-0.107	0.050	0.0343*	-1404.007	6136.554	0.8192	240.406	41.390	<0.0001*	54.556	64.725	0.4001
*H*. *pylori*	-0.096	0.164	0.5576	-22212.603	20006.355	0.2679	-64.679	134.924	0.6321	-91.621	211.046	0.6646
STEC stx1 & stx2	-0.201	0.064	0.0020*	-5795.417	7852.027	0.4611	-72.567	52.976	0.1720	48.500	82.830	0.5587
Cryptosporidium	0.017	0.031	0.5801	143.496	3779.297	0.9697	42.626	28.721	0.1390	6.316	38.985	0.8714
*Cyclospora*	0.009	0.101	0.9301	-12792.821	12445.159	0.3049	58.113	91.815	0.5273	-175.403	128.178	0.1724
*E*. *bieneusi*	0.089	0.049	0.0673	-7183.708	5992.814	0.2317	12.197	44.231	0.7830	-41.989	64.349	0.5146
*Giardia*	0.054	0.021	0.0091*	3249.669	2533.286	0.2007	11.925	18.871	0.5280	72.198	26.394	0.0067*
*Trichuris*	0.001	0.165	0.9960	7909.737	20352.996	0.6979	-25.837	150.156	0.8635	-95.863	209.633	0.6478
Adenovirus 40/41	0.052	0.025	0.0399*	-5678.013	3073.041	0.0658	45.478	23.272	0.0518	6.561	33.448	0.8446
Astrovirus	0.093	0.028	0.0008*	6102.398	3371.487	0.0714	13.766	25.397	0.5883	21.750	36.408	0.5508
Enterovirus	0.029	0.023	0.2015	3258.000	2795.368	0.2449	-47.315	21.170	0.0263*	-20.881	30.004	0.4871
Norovirus GI, GII	0.036	0.021	0.0902	-1707.927	2582.729	0.5090	21.473	19.581	0.2738	31.404	27.746	0.2588
Rotavirus	0.035	0.037	0.3492	-5967.456	4572.710	0.1930	6.533	34.087	0.8482	-36.507	50.040	0.4663
Sapovirus	0.012	0.037	0.7378	-8799.921	4399.301	0.0465*	-34.222	32.823	0.2981	6.039	46.964	0.8978

Note: This regression model has been described in the methods section as Model 2: Biomarker = β_0_ + β_1_ pathogen_1_ + β_2_ pathogen_2_ + … + β_k_pathogen_k_ + β_k+1_ antibiotics + ε. β_k_ is estimated effects of each pathogen obtained via a multiple linear regression model using the levels of biomarkers at 6 months as the dependent variable (continuous) and the presence of each pathogen (categorical) as multiple independent variables. Antibiotic use (categorical variable) was included in the model as a covariate.

***p-value<0.0001

**p-value<0.001

*p-value<0.05

Abbreviations: Flagellin = flic; Immunoglobulin = Ig; alpha glycoprotein = AGP; C-reactive protein = CRP; Myeloperoxidase = MPO; regenerating gene 1 beta = Reg1b

¥Biomarkers were indicative of systemic inflammation (CRP, AGP), bacterial translocation (anti-flic IgA), enteric inflammation (MPO), and intestinal regeneration (Reg1b). The specific biomarkers included in this analysis were chosen because they significantly correlated with increasing pathogen counts at either 6 or 9 months

## Discussion

In the context of EED, the present study highlights the association between enteropathogens and linear growth–an association hypothesized to be mediated through enteric and systemic inflammatory pathway [15]. The key findings of our study are: a) the presence of at least one enteropathogen in fecal samples at 6 and 9 months of age (Table 1) (at least one bacteria, one protozoa and one virus); b) a negative correlation of delta LAZ with observed pathogen at 6 months (Table 2); and c) an association of specific enteropathogens with positive or negative changes in beta estimates of EED biomarkers as outcome variable.

Overall we found that subclinical infection with entropathogen was marginally associated with linear growth. The presence of similar pathogens such as Giardia, Campylobacter and Cryptosporidium also showed substantial negative association in MAL-ED cohort [26].

In our study, we found that *Campylobacter* infection at 6 months of age had a negative effect on future LAZ at nominal significance level and the effect became marginally after FDR adjustment (Table 3). *Campylobacter* infection has been linked to inflammation and disruption of the gut barrier functions [27], and with reduced weight gain in developing nations [28]. Additionally, the GEMS study, which focused on characterizing the burden of diarrheal disease in Asia and Africa, identified *Campylobacter* as an important contributor to the diarrheal disease burden with regional importance in Pakistan, Bangladesh and India [29]. Since the GEMS study focused on diarrheal episodes, it is interesting to observe an association of *Campylobacter* with asymptomatic children with linear growth faltering. We also found that *Campylobacter* infection was associated with an increased expression of fecal MPO and to some extent increase in Reg1b, a marker of enterocyte regeneration. Although both MPO and NEO have previously shown to be highly associated with intestinal inflammation as well as growth failure in children [20, 30, 31]. Our study did not identify any correlation between the presence of *Campylobacter* with NEO per se. In light of prior research, it is clear that *Campylobacter* is a pathogen of emerging importance and is involved in the causal pathway of environmental enteropathy in developing countries.

The presence of Cryptosporidium and *Giardia* at 6 months showed negative decline in LAZ (range -0.3 to -0.5) in the first 18 months of life, which was also supported by other malnourished cohorts [32] [33]. Early infection of children with Cryptosporidium and *Giardia* at 6 months of age is associated with linear growth faltering. The presence of the above pathogens was associated with a significant decline in LAZ (range -0.3 to -0.5) in the first 18 months of life [11, 34]. In the GEMS study, Cryptosporidium infection was ranked among top three pathogens associated with linear growth in children with less-severe diarrhea (LSD) and moderate-to-severe diarrhea (MSD) as per GEMS definition [11, 34]. Additionally, *Giardia* infection has also been associated with intestinal permeability, malabsorption [33], and poor linear growth, as hallmark features of EED [33, 35]. Furthermore, Berkman et al studied the effect of *Giardia* infection on malnutrition and cognitive development in older children, as an important outcome of EED [36] [37].

Among etiologies of diarrheal infection, Norovirus has been identified as an important enteropathogen of malnutrition and growth faltering in the Mal-ED and GEMS studies [29, 38]. Association of Norovirus and malnutrition is well established in murine model [39], which may involve in modulation of tight junctions. In asymptomatic children with non-diarrheal stools, we did not find any significance of Norovirus on growth decline.

Increased pathogen count at 6 months also correlated with increase in anti-flic IgA levels, an activation signal of innate immune response initiated by recognition of PAMPS (bacterial flagellin) by Toll-like receptor 5 (TLR5), which activates downstream signaling pathway of NF-kB regulated pro-inflammatory proteins. Bacterial flagellin is thought to be involved in mucosal damage by targeting the basolateral surface of intestinal cells. [40, 41]. In our cohort, we also found an association of antibodies against Anti-flic IgA with biomarkers of intestinal inflammation and regeneration (MPO and Reg1b) [15]. Anti-flic IgA antibodies are therefore considered a marker of translocation of flagellin-producing bacteria in blood [42].

Reg1b is a marker of enterocyte regeneration [43], which is associated with presence of enterotoxigenic *E*. *coli* (ETEC) and *Campylobacter* infections at 6 and 9 months of age. Reg 1b is a predictor of childhood stunting in Bangladeshi cohort [44]. Reg1β did not show a direct relationship with linear growth faltering previously shown in this cohort [16]

We found that biomarkers of systemic inflammation, CRP, AGP and Ferritin, were not significantly associated with overall burden of pathogens. On stratification, only protozoal infection at 9 months was moderately correlated with inflammatory markers, which indicates the process of cellular damage and activation of immune mechanism. Such an increase in CRP has been reported in travelers in tropics with protozoal infections [45]. In the acute phase of bacterial diarrhea in Egyptian children, CRP was identified as marker of acute inflammation along with Trem-1 and Procalcitonin [46]. The lack of CRP elevation in our study could reflect low frequency of diarrhea in our children and indicates that they had probably passed the acute phase of infection. An early rise in CRP and AGP have been associated with stunting in Zimbabwean infants and, as recently reported, with future stunting in this Pakistani cohort [18, 47]. Furthermore, a recent South Indian study evaluated asymptomatic carriage of enteropathogens in children from two communities to study the effect of the environment on pathogen burden and corresponding changes in inflammatory biomarkers [48]. In comparison to this study, our study had a longitudinal study design for both biological sample collection and anthropometric measurements. We also analyzed a larger panel of pathogens and wider array of systemic/gut inflammatory biomarkers. Our analyses also focused on association of enteropathogen with changes in LAZ and varying levels of biomarkers.

Strengths of our study include: a) longitudinal follow-up with prospective repeated measures of growth; b) use of a highly sensitive platform that has shown superior sensitivity in the detection of enteropathogens as already been utilized in the MAL-ED [23] and GEMS cohorts [11], and c) co-existence of enteropathogens and EED biomarkers spanning gut-specific, systemic, and mucosal inflammatory responses. Our study is limited with a) censoring of data at 18 months of age, thus limiting follow-up beyond 18 months to be able to ascertain the persistence of infection and outcome on long term growth and cognition; b) we evaluated growth faltering as changes in the linear slope of z scores, and thus were limited in using growth faltering at the first 18 months of life as a clinical proxy of EED; and lastly c) we were unable to collect dietary information in this cohort to correlate these important findings and d) multiple comparison of pathogens and biomarker data in regression model lost some of the significance after correction for FDR. Also, the growth modelling did not account for early and later stunting in this cohort.

## Conclusion

In conclusion, childhood infection with increasing numbers of pathogens is associated with an increase in biomarkers of inflammation and intestinal permeability with a decrease in linear growth. Moreover, specific pathogens, such as *Campylobacter*, Cryptosporidium *and Giardia* seem to play a key role in such associations with growth and increase in inflammatory biomarkers which may be involved in the process of EED. With the advent of a combination vaccine against *Shigella* and enterotoxigenic *E*. *coli*, prevention of enteric infections may reduce the risk of enteric infections that seem to play a critical role in the pathogenesis of EED.

## Supporting information

S1 Fig Cumulative Z scores with comorbidity data up to 18 months of age. Descriptive data regarding the length-for-age (LAZ), weight-for-age (WAZ), and weight-for-length (WHZ) Z scores for the Pakistani cohort over the course of the first 18 months of life (scatterplot and left y-axis). Also included are data regarding the mean number of days in each month that children reported diarrhea or acute respiratory infection (ARI), defined as the presence of a cough and/or runny nose (bars, right y-axis).
(JPG)

S2 Fig Bar graph representing frequencies of pathogens detected by Array card. Comparison of the frequencies of bacterial (A), viral (B) and protozoal (C) enteropathogens detected in the cohort A (6 months) and cohort B (9 months).
(PNG)

S1 Table Additional descriptive statistics for the children in the analysis. (DOCX)

S2 Table The association of select enteropathogens at 9 months (n = 271) with levels of specific biomarkers¥ at 9 months. Note: This regression model has been described in the methods section as Model 2: Biomarker = β0 + β1 pathogen1 + β2pathogen2 + … + βnpathogenn + βn+1antibiotics + ε. The β estimates for each pathogen were obtained via a multiple linear regression model using the levels of biomarkers over the first 18 months of life as the dependent variable (continuous) and the presence of each pathogen (categorical) as multiple independent variables. Antibiotic use (categorical variable) was included in the model as a covariate. ***p-value<0.0001; **p-value<0.001; *p-value<0.05 Abbreviations: Flagellin = flic; Immunoglobulin = Ig; alpha glycoprotein = AGP; C-reactive protein = CRP; Myeloperoxidase = MPO; regenerating gene 1 beta = Reg 1b
¥Biomarkers were indicative of systemic inflammation (CRP, AGP), bacterial translocation (anti-flic IgA), enteric inflammation (MPO), and intestinal regeneration (Reg 1b). The specific biomarkers included in this analysis were chosen because they significantly correlated with increasing pathogen counts at either 6 or 9 months.
(DOCX)

## References

[1] Kelly P, Menzies I, Crane R, Zulu I, Nickols C, Feakins R, et al Responses of small intestinal architecture and function over time to environmental factors in a tropical population. The American journal of tropical medicine and hygiene. 2004;70(4):412–9. 15100456

[2] Keusch GT, Denno DM, Black RE, Duggan C, Guerrant RL, Lavery JV, et al Environmental enteric dysfunction: pathogenesis, diagnosis, and clinical consequences. Clinical Infectious Diseases. 2014;59(suppl_4):S207–S12. 25305288 10.1093/cid/ciu485 PMC4481570

[3] Campbell D, Elia M, Lunn P. Growth faltering in rural Gambian infants is associated with impaired small intestinal barrier function, leading to endotoxemia and systemic inflammation. The Journal of nutrition. 2003;133(5):1332–8. 10.1093/jn/133.5.1332 12730419

[4] Guerrant RL, DeBoer MD, Moore SR, Scharf RJ, Lima AA. The impoverished gut—a triple burden of diarrhoea, stunting and chronic disease. Nature Reviews Gastroenterology and Hepatology. 2013;10(4):220–9. 10.1038/nrgastro.2012.239 23229327 PMC3617052

[5] Dewey KG, Adu‐Afarwuah S. Systematic review of the efficacy and effectiveness of complementary feeding interventions in developing countries. Maternal & child nutrition. 2008;4(s1):24–85. 18289157 10.1111/j.1740-8709.2007.00124.x PMC6860813

[6] Gilmartin AA, Petri WA. Exploring the role of environmental enteropathy in malnutrition, infant development and oral vaccine response. Phil Trans R Soc B. 2015;370(1671):20140143 10.1098/rstb.2014.0143 25964455 PMC4527388

[7] Kosek MN, Mduma E, Kosek PS, Lee GO, Svensen E, Pan WK, et al Plasma Tryptophan and the Kynurenine–Tryptophan Ratio are Associated with the Acquisition of Statural Growth Deficits and Oral Vaccine Underperformance in Populations with Environmental Enteropathy. The American journal of tropical medicine and hygiene. 2016;95(4):928–37. 10.4269/ajtmh.16-0037 27503512 PMC5062803

[8] Korpe PS, Petri WA. Environmental enteropathy: critical implications of a poorly understood condition. Trends in molecular medicine. 2012;18(6):328–36. 10.1016/j.molmed.2012.04.007 22633998 PMC3372657

[9] Ramakrishna B, Venkataraman S, Mukhopadhya A. Tropical malabsorption. Postgraduate medical journal. 2006;82(974):779–87. 10.1136/pgmj.2006.048579 17148698 PMC2653921

[10] Bhutta ZA, Ahmed T, Black RE, Cousens S, Dewey K, Giugliani E, et al What works? Interventions for maternal and child undernutrition and survival. The lancet. 2008;371(9610):417–40. 10.1016/S0140-6736(07)61693-6 18206226

[11] Liu J, Platts-Mills JA, Juma J, Kabir F, Nkeze J, Okoi C, et al Use of quantitative molecular diagnostic methods to identify causes of diarrhoea in children: a reanalysis of the GEMS case-control study. Lancet. 2016;388(10051):1291–301. Epub 2016/09/28. 10.1016/S0140-6736(16)31529-X 27673470 PMC5471845

[12] Investigators M-EN. The MAL-ED study: a multinational and multidisciplinary approach to understand the relationship between enteric pathogens, malnutrition, gut physiology, physical growth, cognitive development, and immune responses in infants and children up to 2 years of age in resource-poor environments. Clinical infectious diseases: an official publication of the Infectious Diseases Society of America. 2014;59:S193. 25305287 10.1093/cid/ciu653

[13] Syed S, Ali A, Duggan C. Environmental enteric dysfunction in children: a review. Journal of pediatric gastroenterology and nutrition. 2016;63(1):6 10.1097/MPG.0000000000001147 26974416 PMC4920693

[14] Wennerås C, Erling V. Prevalence of enterotoxigenic Escherichia coli-associated diarrhoea and carrier state in the developing world. Journal of Health, Population and Nutrition. 2004:370–82. 15663170

[15] Syed S, Iqbal NT, Sadiq K, Ma JZ, Akhund T, Xin W, et al Serum anti-flagellin and anti-lipopolysaccharide immunoglobulins as predictors of linear growth faltering in Pakistani infants at risk for environmental enteric dysfunction. PLoS One. 2018;13(3):e0193768 Epub 2018/03/07. 10.1371/journal.pone.0193768 29509790 PMC5839587

[16] Iqbal NT, Sadiq K, Syed S, Akhund T, Umrani F, Ahmed S, et al Promising Biomarkers of Environmental Enteric Dysfunction: A Prospective Cohort study in Pakistani Children. Sci Rep. 2018;8(1):2966 Epub 2018/02/16. 10.1038/s41598-018-21319-8 29445110 PMC5813024

[17] Sunil Syed SY; Jeremy Herrmann; Anne Sailer; Kamran Sadiq; Najeeha Iqbal; Furqan Kabir; Kumail Ahmed; Shahida Qureshi; Sean Moore; Jerrold Turner; Asad Ali. Environmental Enteropathy in Undernourished Pakistani Children: Clinical and Histomorphometric Analyses. American Journal of Tropical Medicine & Hygiene. 2018 10.4269/ajtmh.17-0306 29611507 PMC6086170

[18] Iqbal NT, Sadiq K, Syed S, Akhund T, Umrani F, Ahmed S, et al Promising Biomarkers of Environmental Enteric Dysfunction: A Prospective Cohort study in Pakistani Children. Scientific reports. 2018;8(1):2966 10.1038/s41598-018-21319-8 29445110 PMC5813024

[19] Estívariz CF, Griffith DP, Luo M, Szeszycki EE, Bazargan N, Dave N, et al Efficacy of parenteral nutrition supplemented with glutamine dipeptide to decrease hospital infections in critically ill surgical patients. Journal of Parenteral and Enteral Nutrition. 2008;32(4):389–402. 10.1177/0148607108317880 18596310 PMC3062504

[20] Kosek M, Haque R, Lima A, Babji S, Shrestha S, Qureshi S, et al Fecal markers of intestinal inflammation and permeability associated with the subsequent acquisition of linear growth deficits in infants. The American journal of tropical medicine and hygiene. 2013;88(2):390–6. 10.4269/ajtmh.2012.12-0549 23185075 PMC3583335

[21] Liu J, Gratz J, Amour C, Kibiki G, Becker S, Janaki L, et al A laboratory-developed TaqMan Array Card for simultaneous detection of 19 enteropathogens. J Clin Microbiol. 2013;51(2):472–80. 10.1128/JCM.02658-12 23175269 PMC3553916

[22] Liu J, Gratz J, Amour C, Nshama R, Walongo T, Maro A, et al Optimization of quantitative PCR methods for enteropathogen detection. PloS one. 2016;11(6):e0158199 10.1371/journal.pone.0158199 27336160 PMC4918952

[23] Liu J, Kabir F, Manneh J, Lertsethtakarn P, Begum S, Gratz J, et al Development and assessment of molecular diagnostic tests for 15 enteropathogens causing childhood diarrhoea: a multicentre study. Lancet Infect Dis. 2014;14(8):716–24. Epub 2014/07/16. 10.1016/S1473-3099(14)70808-4 . 25022434

[24] de Onis M, Onyango AW, Borghi E, Siyam A, Nishida C, Siekmann J. Development of a WHO growth reference for school-aged children and adolescents. Bulletin of the World Health Organization. 2007;85(9):660–7. 10.2471/BLT.07.043497 18026621 PMC2636412

[25] WHO. WHO child growth standards SAS macro (version 3.2.2) Geneva (Switzerland): World Health Organization Available from: http://www.who.int/childgrowth/software/readme_sas.pdf.

[26] Rogawski ET, Liu J, Platts-Mills JA, Kabir F, Lertsethtakarn P, Siguas M, et al Use of quantitative molecular diagnostic methods to investigate the effect of enteropathogen infections on linear growth in children in low-resource settings: longitudinal analysis of results from the MAL-ED cohort study. Lancet Glob Health. 2018;6(12):e1319–e28. Epub 2018/10/06. 10.1016/S2214-109X(18)30351-6 30287125 PMC6227248

[27] Black RE, Levine MM, Clements ML, Hughes TP, Blaser MJ. Experimental Campylobacter jejuni infection in humans. Journal of infectious diseases. 1988;157(3):472–9. 10.1093/infdis/157.3.472 3343522

[28] Lee G, Pan W, Yori PP, Olortegui MP, Tilley D, Gregory M, et al Symptomatic and asymptomatic Campylobacter infections associated with reduced growth in Peruvian children. PLoS neglected tropical diseases. 2013;7(1):e2036 10.1371/journal.pntd.0002036 23383356 PMC3561130

[29] Kotloff KL, Nataro JP, Blackwelder WC, Nasrin D, Farag TH, Panchalingam S, et al Burden and aetiology of diarrhoeal disease in infants and young children in developing countries (the Global Enteric Multicenter Study, GEMS): a prospective, case-control study. The Lancet. 2013;382(9888):209–22. 10.1016/S0140-6736(13)60844-2 23680352

[30] Campbell DI, McPhail G, Lunn PG, Elia M, Jeffries DJ. Intestinal inflammation measured by fecal neopterin in Gambian children with enteropathy: association with growth failure, Giardia lamblia, and intestinal permeability. Journal of pediatric gastroenterology and nutrition. 2004;39(2):153–7. 10.1097/00005176-200408000-00005 15269619

[31] Naylor C, Lu M, Haque R, Mondal D, Buonomo E, Nayak U, et al Environmental enteropathy, oral vaccine failure and growth faltering in infants in Bangladesh. EBioMedicine. 2015;2(11):1759–66. 10.1016/j.ebiom.2015.09.036 26870801 PMC4740306

[32] Korpe PS, Valencia C, Haque R, Mahfuz M, McGrath M, Houpt E, et al Epidemiology and Risk Factors for Cryptosporidiosis in Children From 8 Low-income Sites: Results From the MAL-ED Study. Clin Infect Dis. 2018;67(11):1660–9. Epub 2018/04/28. 10.1093/cid/ciy355 29701852 PMC6233690

[33] Rogawski ET, Bartelt LA, Platts-Mills JA, Seidman JC, Samie A, Havt A, et al Determinants and impact of giardia infection in the first 2 years of life in the MAL-ED birth cohort. Journal of the Pediatric Infectious Diseases Society. 2017;6(2):153–60. 10.1093/jpids/piw082 28204556 PMC5907871

[34] Sow SO, Muhsen K, Nasrin D, Blackwelder WC, Wu Y, Farag TH, et al The burden of Cryptosporidium diarrheal disease among children< 24 months of age in moderate/high mortality regions of Sub-Saharan Africa and South Asia, utilizing data from the Global Enteric Multicenter Study (GEMS). PLoS neglected tropical diseases. 2016;10(5):e0004729 10.1371/journal.pntd.0004729 27219054 PMC4878811

[35] Donowitz JR, Alam M, Kabir M, Ma JZ, Nazib F, Platts-Mills JA, et al A prospective longitudinal cohort to investigate the effects of early life giardiasis on growth and all cause diarrhea. Clinical Infectious Diseases. 2016;63(6):792–7. 10.1093/cid/ciw391 27313261 PMC4996141

[36] Berkman DS, Lescano AG, Gilman RH, Lopez SL, Black MM. Effects of stunting, diarrhoeal disease, and parasitic infection during infancy on cognition in late childhood: a follow-up study. The Lancet. 2002;359(9306):564–71. 10.1016/S0140-6736(02)07744-9 11867110

[37] Syed S, Ali A, Duggan C. Environmental Enteric Dysfunction in Children. J Pediatr Gastroenterol Nutr. 2016;63(1):6–14. 10.1097/MPG.0000000000001147 26974416 PMC4920693

[38] Rouhani S, Peñataro Yori P, Paredes Olortegui M, Siguas Salas M, Rengifo Trigoso D, Mondal D, et al Norovirus infection and acquired immunity in 8 countries: results from the MAL-ED study. Clinical Infectious Diseases. 2016;62(10):1210–7. 10.1093/cid/ciw072 27013692 PMC4845786

[39] Troeger H, Loddenkemper C, Schneider T, Schreier E, Epple HJ, Zeitz M, et al Structural and functional changes of the duodenum in human norovirus infection. Gut. 2009;58(8):1070–7. 10.1136/gut.2008.160150 . 19036950

[40] Gewirtz AT, Navas TA, Lyons S, Godowski PJ, Madara JL. Cutting edge: bacterial flagellin activates basolaterally expressed TLR5 to induce epithelial proinflammatory gene expression. The Journal of Immunology. 2001;167(4):1882–5. 10.4049/jimmunol.167.4.1882 11489966

[41] Gewirtz AT, Simon PO Jr, Schmitt CK, Taylor LJ, Hagedorn CH, O’Brien AD, et al Salmonella typhimurium translocates flagellin across intestinal epithelia, inducing a proinflammatory response. Journal of Clinical Investigation. 2001;107(1):99 10.1172/JCI10501 11134185 PMC198545

[42] Thorpe C, Hurley BP, Lincicome LL, Jacewicz MS, Keusch GT, Acheson DW. Shiga toxins stimulate secretion of interleukin-8 from intestinal epithelial cells. Infection and Immunity. 1999;67(11):5985–93. 10531258 10.1128/iai.67.11.5985-5993.1999 PMC96984

[43] Shinozaki S, Nakamura T, Iimura M, Kato Y, Iizuka B, Kobayashi M, et al Upregulation of Reg 1α and GW112 in the epithelium of inflamed colonic mucosa. Gut. 2001;48(5):623–9. 10.1136/gut.48.5.623 11302958 PMC1728274

[44] Peterson KM, Buss J, Easley R, Yang Z, Korpe PS, Niu F, et al REG1B as a predictor of childhood stunting in Bangladesh and Peru–. The American of Clinical Nutrition. 2013;97(5):1129–33. 10.3945/ajcn.112.048306 PMC3628379 23553156

[45] Herbinger K-H, Hanus I, Schunk M, Beissner M, von Sonnenburg F, Löscher T, et al Elevated Values of C-Reactive Protein Induced by Imported Infectious Diseases: A Controlled Cross-Sectional Study of 11,079 Diseased German Travelers Returning from the Tropics and Subtropics. The American journal of tropical medicine and hygiene. 2016;95(4):938–44. 10.4269/ajtmh.16-0387 27527624 PMC5062804

[46] Al-Asy HM, Gamal RM, Albaset AMA, Elsanosy MG, Mabrouk MM. New diagnostic biomarker in acute diarrhea due to bacterial infection in children. International Journal of Pediatrics and Adolescent Medicine. 2017;4(2):75–80. 10.1016/j.ijpam.2016.12.004 30805506 PMC6372495

[47] Prendergast AJ, Rukobo S, Chasekwa B, Mutasa K, Ntozini R, Mbuya MN, et al Stunting is characterized by chronic inflammation in Zimbabwean infants. PloS one. 2014;9(2):e86928 10.1371/journal.pone.0086928 24558364 PMC3928146

[48] Praharaj I, Revathy R, Bandyopadhyay R, Benny B, KO MA, Liu J, et al Enteropathogens and Gut Inflammation in Asymptomatic Infants and Children in Different Environments in Southern India. The American journal of tropical medicine and hygiene. 2018;98(2):576–80. 10.4269/ajtmh.17-0324 29231154 PMC5929183

