# Supplementary figures and images for: Pathobiome driven gut inflammation in Pakistani children with Environmental Enteric Dysfunction
Source: PLoS One. 2019 Aug 23;14(8):e0221095. doi: 10.1371/journal.pone.0221095 (PMC6707605; doi:10.1371/journal.pone.0221095)

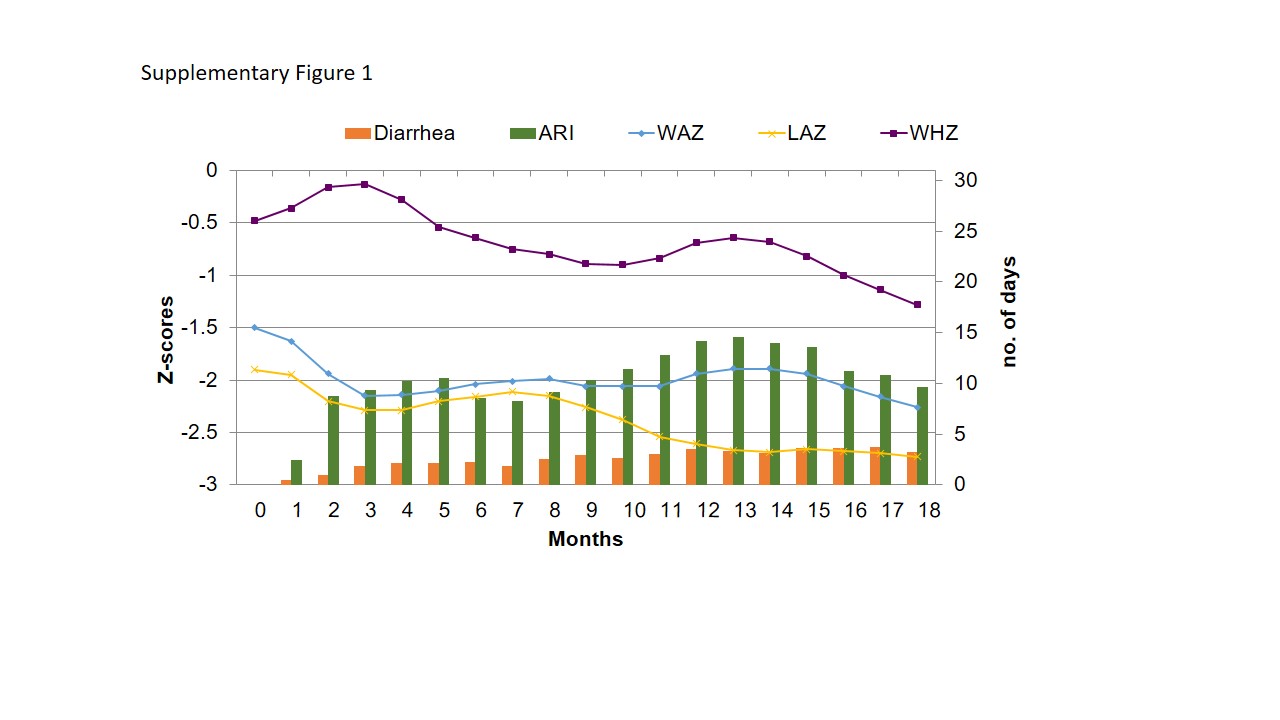

Supplement: S1 Fig — Descriptive data regarding the length-for-age (LAZ), weight-for-age (WAZ), and weight-for-length (WHZ) Z scores for the Pakistani cohort over the course of the first 18 months of life (scatterplot and left y-axis). Also included are data regarding the mean number of days in each month that children reported diarrhea or acute respiratory infection (ARI), defined as the presence of a cough and/or runny nose (bars, right y-axis). (JPG) [file pone.0221095.s001.jpg]

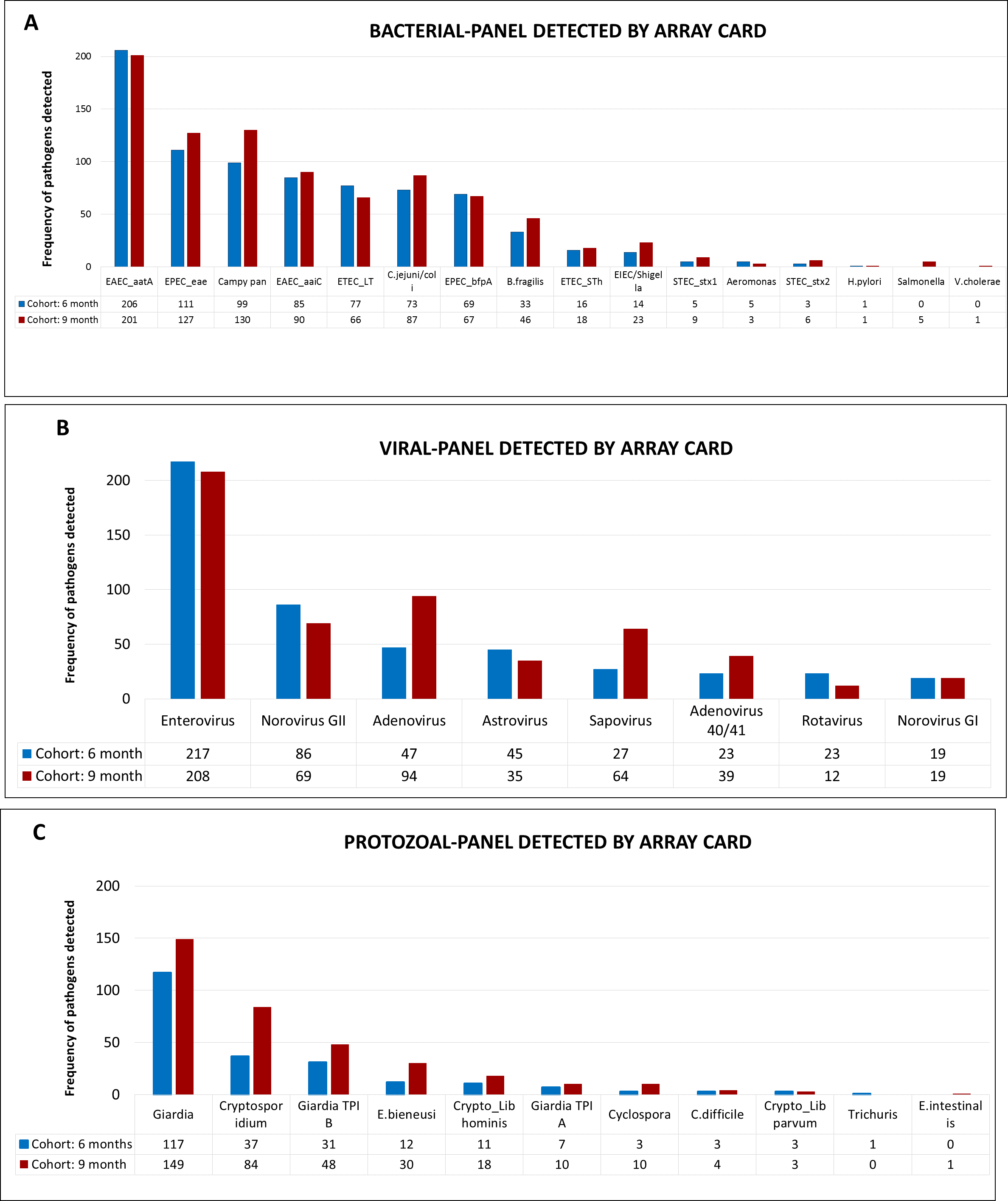

Supplement: S2 Fig — Comparison of the frequencies of bacterial (A), viral (B) and protozoal (C) enteropathogens detected in the cohort A (6 months) and cohort B (9 months). (PNG) [file pone.0221095.s002.png]
