# Supplementary material for: Pathobiome driven gut inflammation in Pakistani children with Environmental Enteric Dysfunction
Source: PLoS One. 2019 Aug 23;14(8):e0221095. doi: 10.1371/journal.pone.0221095 (PMC6707605; doi:10.1371/journal.pone.0221095)
Supplement: S1 Table — (DOCX) [file pone.0221095.s003.docx]

**S1 Table. Descriptive statistics of the mothers and children in the analysis.**

| **Baseline characteristics of mothers** | |
| --- | --- |
| **Variable** | **Mean ± SD** |
| Maternal age (year) | 30.0 ± 3.9 |
| Maternal Educational level | No education (86.8%) Primary (8.7%) Secondary (2.9%) Higher Secondary (0.5%) Graduation (0.5%) |
| **Baseline characteristics of children** | |
| HAZ at enrollment | -1.91 ± 1.38 |
| WAZ at enrollment | -1.52 ± 1.18 |
| HAZ at 18 month | -2.19 ± 1.18 |
| WAZ at 18 month | -2.68 ± 1.15 |
| Gestational age (week) | 36.6 ± 1.2 |
| Preterm birth n (%) | 174 (64%) |
| Birth weight (grams) | 2639 ± 505 |
| Use of antibiotics up to 6 month (Yes) n(%) | 31 (11%) |
| Use of antibiotics up to 9 month (Yes) n(%) | 34 (13%) |
