# Supplementary material for: Pathobiome driven gut inflammation in Pakistani children with Environmental Enteric Dysfunction
Source: PLoS One. 2019 Aug 23;14(8):e0221095. doi: 10.1371/journal.pone.0221095 (PMC6707605; doi:10.1371/journal.pone.0221095)
Supplement: S2 Table — Note: This regression model has been described in the methods section as Model 2: Biomarker = β0 + β1 pathogen1 + β2pathogen2 + … + βnpathogenn + βn+1antibiotics + ε. The β estimates for each pathogen were obtained via a multiple linear regression model using the levels of biomarkers over the first 18 months of life as the dependent variable (continuous) and the presence of each pathogen (categorical) as multiple independent variables. Antibiotic use (categorical variable) was included in the model as a covariate. ***p-value<0.0001; **p-value<0.001; *p-value<0.05 Abbreviations: Flagellin = flic; Immunoglobulin = Ig; alpha glycoprotein = AGP; C-reactive protein = CRP; Myeloperoxidase = MPO; regenerating gene 1 beta = Reg 1b ¥Biomarkers were indicative of systemic inflammation (CRP, AGP), bacterial translocation (anti-flic IgA), enteric inflammation (MPO), and intestinal regeneration (Reg 1b). The specific biomarkers included in this analysis were chosen because they significantly correlated with increasing pathogen counts at either 6 or 9 months. (DOCX) [file pone.0221095.s004.docx]

**Supporting information**

**S2 Table . The association of select enteropathogens at 9 months (n = 271) with levels of specific biomarkers^¥^ at 9 months.**

|  | **Biomarkers at 9 months** | | | | | | | | | | | |
| --- | --- | --- | --- | --- | --- | --- | --- | --- | --- | --- | --- | --- |
|  | **CRP** | | | **AGP** | | | **Reg1b Serum** | | | **Reg1b fecal** | | |
| **Enteropathogens** | **β** | **SE** | **p-value** | **β** | **SE** | **p-value** | **β** | **SE** | **p-value** | **β** | **SE** | **p-value** |
| *Aeromonas* | 0.8 | 0.8 | 0.35 | -0.4 | 33.6 | 0.99 | 218.4 | 144.0 | 0.13 | 98.8 | 182.6 | 0.59 |
| *Bacteroides fragilis* | 0.3 | 0.2 | 0.15 | -0.1 | 8.5 | 0.99 | -41.5 | 35.9 | 0.25 | -50.2 | 44.5 | 0.26 |
| *C. difficile* | -0.1 | 0.6 | 0.83 | -8.3 | 24.1 | 0.73 | -28.0 | 103.6 | 0.79 | -21.2 | 131.0 | 0.87 |
| *Campylobacter* | 0.09 | 0.1 | 0.53 | 6.5 | 6.0 | 0.28 | 73.0 | 25.6 | 0.0047* | 42.8 | 32.4 | 0.19 |
| EAEC | 0.2 | 0.2 | 0.22 | -4.9 | 7.4 | 0.51 | 16.3 | 31.3 | 0.60 | -29.2 | 39.3 | 0.46 |
| EIEC *Shigella* toxin | 0.2 | 0.3 | 0.43 | 21.9 | 10.5 | 0.037* | 21.7 | 44.2 | 0.62 | 9.0 | 56.9 | 0.87 |
| EPEC | -0.08 | 0.2 | 0.58 | -0.7 | 6.3 | 0.91 | 37.8 | 26.5 | 0.16 | 50.5 | 33.3 | 0.13 |
| ETEC LT_STp | 0.05 | 0.2 | 0.77 | -1.5 | 7.0 | 0.83 | -8.8 | 29.7 | 0.77 | 47.6 | 37.2 | 0.20 |
| ETEC STh | -0.3 | 0.3 | 0.31 | -7.6 | 11.8 | 0.52 | -14.0 | 49.7 | 0.78 | -21.9 | 62.8 | 0.73 |
| *H. pylori* | 0.4 | 1.1 | 0.71 | 55.3 | 47.1 | 0.24 | -137.2 | 202.3 | 0.50 | 276.6 | 256.4 | 0.28 |
| STEC 12 | -0.6 | 0.3 | 0.09 | -27.6 | 13.7 | 0.046* | -47.3 | 58.8 | 0.42 | 17.0 | 77.2 | 0.83 |
| *Cryptosporidium* | 0.2 | 0.2 | 0.33 | 15.4 | 6.6 | 0.020* | 8.0 | 28.3 | 0.78 | 37.0 | 34.7 | 0.29 |
| *Cyclospora* | -0.6 | 0.4 | 0.13 | -2.3 | 16.0 | 0.89 | 18.0 | 70.6 | 0.80 | -108.6 | 83.1 | 0.19 |
| *E. bieneusi* | 0.09 | 0.2 | 0.68 | -3.4 | 8.8 | 0.70 | 17.2 | 38.6 | 0.66 | -75.6 | 49.9 | 0.13 |
| *Giardia* | 0.2 | 0.1 | 0.25 | 5.6 | 5.8 | 0.34 | 0.5 | 25.4 | 0.99 | 47.6 | 31.3 | 0.13 |
| *Trichuris* | -- | -- | -- | -- | -- | -- | -- | -- | -- | -- | -- | -- |
| Adenovirus | -0.1 | 0.1 | 0.44 | 2.4 | 6.0 | 0.69 | -4.4 | 25.6 | 0.86 | 57.3 | 32.2 | 0.08 |
| Astrovirus | -0.3 | 0.2 | 0.18 | -14.1 | 8.8 | 0.11 | 16.1 | 37.7 | 0.67 | 17.1 | 47.5 | 0.72 |
| Enterovirus | 0.04 | 0.2 | 0.83 | 1.5 | 6.9 | 0.83 | 14.5 | 29.2 | 0.62 | 37.3 | 36.7 | 0.31 |
| Norovirus 12 | -0.2 | 0.2 | 0.13 | -4.7 | 6.3 | 0.46 | -14.4 | 26.8 | 0.59 | -36.0 | 33.5 | 0.28 |
| Rotavirus | 0.04 | 0.3 | 0.91 | 0.3 | 14.0 | 0.98 | -12.1 | 60.1 | 0.84 | -98.7 | 75.7 | 0.19 |
| Sapovirus | -0.3 | 0.2 | 0.08 | -2.8 | 6.6 | 0.67 | 68.3 | 28.2 | 0.016* | 13.8 | 35.3 | 0.70 |
